# A multiplex targeted NGS panel for identifying pathogens in canine neurological and reproductive diseases

**DOI:** 10.3389/fcimb.2025.1591145

**Published:** 2025-08-01

**Authors:** Jobin Jose Kattoor, Eman Anis, Nelly O. Elshafie, Rebecca P. Wilkes

**Affiliations:** ^1^ Department of Infectious Diseases and Microbiology, School of Public Health, University of Pittsburgh, Pittsburgh, PA, United States; ^2^ Willie M. Reed Laboratory, Indiana Animal Disease Diagnostic Laboratory, Purdue University, West Lafayette, IN, United States; ^3^ Department of Pathobiology, Pennsylvania Animal Diagnostic Laboratory System-New Bolton Center, University of Pennsylvania School of Veterinary Medicine, Kennett Square, PA, United States; ^4^ Department of Veterinary Science and Veterinary Diagnostic Laboratory, University of Kentucky, Lexington, KY, United States

**Keywords:** targeted NGS, ion torrent, canine reproductive disease, canine neurological disease, molecular diagnostics

## Abstract

**Introduction:**

Multiple pathogens can infect the canine reproductive and central nervous systems, including organisms that are zoonotic, such as *Brucella canis*, pathogenic *Leptospira* spp., *Anaplasma phagocytophilum*, *Histoplasma capsulatum*, and *Blastomyces dermatitidis*. In this study, we developed a targeted next-generation sequencing (tNGS) panel to identify common infectious agents related to neurologic and reproductive disease in canines while incorporating less common zoonotic agents into a single test.

**Methods:**

Primer pools were developed to detect 34 pathogens and used in two multiplex PCR assays, which were combined prior to library preparation and sequencing using Ion Torrent technology. A feasibility study was performed with known positive clinical samples, bacterial isolates, or synthetic DNA (gBlocks) spiked into nucleic acids from negative canine clinical samples.

**Results:**

Of the 34 organisms included in the panel, 33 were detectable. Some primer sets were not specific for the intended target organism, based on BLAST analysis (NCBI) of the obtained sequences. Compared to real-time PCR assays, pathogens could be detected at Ct values from 33-38, depending on the pathogen, and at approximately 100–1000 copies based on gBlock testing. A total of 76 samples (39 positive and 40 negative) were tested, representing neurological and reproductive samples. Diagnostic sensitivity and specificity for the assay were calculated as 89% and 98%, respectively. The tNGS assay had the added benefit of strain-typing Canine distemper virus and Canine parvovirus-2 in the positive samples. For reproducibility, a blinded panel was tested by our laboratory and another laboratory using the same tNGS protocol where the assay had an agreement for 16 out of 18 samples with a Cohen’s kappa value of 0.77, indicating high reproducibility.

**Discussion:**

The tNGS assay was not as sensitive as real-time PCR assays, which is a known limitation of this method, and was evident based on the diagnostic sensitivity testing. However, the comprehensive nature of this assay is beneficial for syndromic testing.

## 1 Introduction

A wide array of organisms, including viruses (Akita et al., 1994; Tatum et al., 1999; Benetka et al., 2006), bacteria (Radaelli and Platt, 2002; KENT, 2012; Rylander et al., 2022), protozoa, parasites (Gerhold et al., 2014), fungi (Lavely and Lipsitz, 2005) and prions (Wyatt et al., 1991) can cause infectious neurological diseases in pet animals. Hospitalizations due to neurological disease constitute approximately 7.7-12.7% of total cases presented to the pet hospitals (Gonçalves et al., 2022). Comprehensive studies in European dogs by researchers reported infectious causes in 10-20% of total neurological disease hospitalizations (Fluehmann et al., 2006; Gonçalves et al., 2022). Often, neurologic diseases in pet animals are either underdiagnosed or undiagnosed (Rand et al., 1994; Radaelli and Platt, 2002). Retrospective studies on the etiological agents of neurologic diseases highlight frequent misdiagnoses, where a definitive diagnosis was achieved in only 30-40% of the total cases (Bradshaw et al., 2004). The polymicrobial etiology of neurological disease in dogs is also reported in the literature for infectious etiology of the disease in dogs (Schwab et al., 2007).

Similarly, canine reproductive disorders are also multifaceted. Most agents of canine reproductive diseases are endogenous and seen throughout the reproductive cycle (Watts et al., 1996). There are exceptions in the endogenous origin of reproductive disease where *Brucella canis* is considered one of the major pathogens causing reproductive disease and discospondylitis in canines (Buhmann et al., 2019). Neurological and reproductive disease conditions are often linked to age, hormonal influence, immune status, or other underlying disease conditions of the animal (Niskanen and Thrusfield, 1998; Fukuda, 2001). Although the majority of reproductive diseases are linked to bacterial agents (Paudel et al., 2023), several viral pathogens, including canine herpesvirus1, canine parvovirus 1, etc., also cause infertility in dogs (Truyen et al., 1996; Evermann et al., 2011).

The difficulty of obtaining affected tissue samples often impacts the definitive diagnosis of neurologic disease. With the advancement of molecular techniques, including PCR and sequencing, the accuracy of diagnosis has improved significantly (Barber et al., 2022). However, performing multiple tests to identify diseases with multiple etiological agents can result in significantly higher costs and delays in diagnosis and treatment.

The overarching goal of this study was to develop and standardize a targeted next-generation sequencing (tNGS) assay for detecting pathogens associated with neurological and reproductive diseases in canines. For syndromic testing for diseases with multiple causative agents of disease, using targeted next-generation sequencing with limited turnaround time provides the clinician a more efficient and comprehensive diagnostic approach, compared to running multiple individual tests.

## 2 Materials and methods

### 2.1 Primer design for AmpliSeq^®^ strategy

An array of primers targeting multiple regions in the genomes of 34 different canine neurologic and reproductive pathogens (**Table 1**) was synthesized concertedly with the Agriseq Bioinformatics team (Thermo Fisher Scientific, USA). Multiple primer sets were included for each pathogen, and the panel also included primers for virulence factors for extraintestinal pathogenic *E. coli* (ExPEC). Some of the canine pathogen primers were validated in a previous study (Kattoor et al., 2022). Altogether, the assay includes 672 primers (data not shown), which were divided into two primer pools to obtain maximum amplification of the target regions in the pathogens under study and to minimize the primer interactions. This assay includes primers for additional organisms outside of this study, including ones we previously evaluated for vector-borne pathogens (Kattoor et al., 2022) and for feline respiratory pathogens (Kattoor et al., 2024). Primers were designed based on the highly conserved regions within the genomes of the organisms of interest as well as areas for some pathogens that allow for genotyping (Canine distemper virus and Canine parvovirus 2). Targeted areas of the genomic sequences of the pathogens under study in FASTA format and the target regions of the primers in BED format were stored in the Torrent suite software (TSS, Thermo Fisher Scientific, USA) for downstream processing of the sequenced reads. The FASTA and BED files are provided as **Supplementary Materials**. Primer pools are available from Thermo Fisher Scientific by emailing AgriSeqOrders@thermofisher.com - design number WGAG21047_PRD_CFPv02.

**Table 1:** Pathogenic agents, source of sample, and confirmatory tests performed for the samples used for feasibility and specificity testing of the developed assay.

	Etiological agent	Source	Confirmatory test
Bacteria	*Streptococcus dysgalactiae subsp equisimilis*	gBlock	-
	*Streptococcus equi subsp zooepidemicus*	Clinical isolate	qPCR
	*Klebsiella* spp.	Clinical isolate	qPCR
	*Borrelia burgdorferi*	gBlock	–
	*Staphylococcus aureus*	ATCC isolate	qPCR
	*Leptospira* spp.	Clinical sample (Ct value 20.26)	qPCR
	*Rickettsia* spp.	Clinical sample	tNGS
	*Anaplasma* spp.	Clinical sample	tNGS
	*Erhlichia* spp.	Clinical sample	tNGS
	*Mycoplasma canis*	Clinical sample (Ct value 30.25)	qPCR
	*Mycoplasma cynos*	Clinical sample (Ct value 19.16)	qPCR
	*Campylobacter jejuni*	Clinical isolate	qPCR
	*Salmonella* spp.	Clinical isolate	qPCR
	*Brucella canis*	gBlock	–
	*E. coli*	Clinical isolates (ExPEC)- one containing cnf 1 and one containing cnf 2 virulence factors	Whole genome sequencing
	*Actinomyces* spp.	gBlock	–
	*Pasteurella multocida*	Clinical sample (Ct value 28.62)	qPCR
Virus	Canine distemper Virus	Clinical sample (Ct value 29.69, genotype Am-3)	qPCR
	Canine adenovirus-1	Clinical sample (Ct value 33.21)	qPCR
	Eastern Equine Encephalitis Virus	Clinical sample (Ct value 25.13)	qPCR
	West Nile Virus	Clinical sample (Ct value 15.32)	qPCR
	Bluetongue Virus	Clinical sample (Ct value 21.02, serotype 10)	qPCR
	Rabies Virus	Clinical sample (Ct value 30.18, skunk rabies)	qPCR
	Canine herpesvirus-1	Clinical sample (Ct value 19.23)	qPCR
	Canine parvovirus-1	gBlock	–
	Canine parvovirus-2	Clinical sample, Ct value 17.21 (genotype 2a)	qPCR
Protozoa	*Toxoplasma gondii*	Clinical sample (Ct value 22.83)	qPCR
	*Neospora caninum*	Clinical sample (Ct value 30.51)	qPCR
Fungi	*Cryptococcus neoformans*	gBlock	–
	*Blastomyces dermatidis*	gBlock/Clinical sample	Histology
	*Histoplasma capsulatum*	gBlock	–
	*Coccidioides immitis/ hughesi*	gBlock	–
	*Aspergillus* spp.	gBlock	–
	*Fusarium* spp.	gBlock	–

The confirmatory tests employed were real-time PCR (qPCR), a previously developed targeted next-generation sequencing panel (tNGS (Kattoor et al., 2022), or histology.

### 2.2 Sample, library preparation, and sequencing

For protocol validation, we used clinical samples previously submitted to the Willie M. Reed Laboratory, Animal Disease Diagnostic Laboratory (ADDL) for real-time PCR (qPCR) or for our previously developed vector-borne pathogen tNGS panel (Kattoor et al., 2022). We also used clinical isolates (identified by MALDI-TOF in the ADDL bacteriology laboratory or by whole genome sequencing), ATCC isolates, or synthetic DNA (gBlocks, Integrated DNA Technologies, USA) representing the pathogen targeted region, which were spiked into negative canine samples. The gBlocks were used when a clinical sample containing the targeted pathogen could not be obtained. The source of the samples used in the study is shown in **Table 1**. Nucleic acids were extracted using the MagMAX™ CORE Nucleic Acid Purification Kit (Thermo Fisher Scientific, USA) on a KingFisher Flex System (Thermo Fisher Scientific, USA). The canine matrix used for spiking synthetic DNA was tested for the absence of any pathogens under study using validated qPCR reactions (ADDL, Purdue University).

Sequencing library preparation was performed semi-automatically on an Ion Chef™ Instrument (Thermo Fisher Scientific, USA). Briefly, 10µL of cDNA was prepared from the samples mentioned above using the NGS Reverse Transcription Kit (Thermo Fisher Scientific, USA). For the gBlocks, a dilution corresponding to 10,000 genome equivalents was spiked into negative canine nucleic acid and used to prepare the cDNA. The targeted PCR assay, followed by automated library preparation, was performed on the Ion Chef using the Ion AmpliSeq™ Kit for Chef DL8 (Thermo Fisher Scientific, USA), according to the manufacturer’s instructions. 150 µL of each primer pool (2x concentration) were used for the multiplex PCR, and up to 8 samples per run were used. The automated library preparation generates a 100 pM pool of up to 8 individually barcoded samples. Sequencing was performed on an Ion 530 chip (Thermo Fisher Scientific), with up to 4 library preps of 8 samples each, for a total of 32 samples per chip. Chip loading was performed on the Ion Chef using the Ion 510™ & Ion 520™ & Ion 530™ Kit – Chef (Thermo Fisher Scientific), using fifty microliters total volume, which included an equal amount of each pooled library. The loaded chip was then sequenced on an Ion GeneStudio™ S5 System (Thermo Fisher Scientific, USA). Approximately 500,000 reads per sample were produced. Quality control, barcode-demultiplexing, and mapping to the FASTA file were performed with Torrent Suite Software (TSS) v. 5.16.1 on the Ion Torrent Server. Generated BAM files were evaluated using Geneious Prime v 2023.2.1 (https://www.geneious.com/features/prime).

### 2.3 Feasibility, analytical sensitivity and specificity of the assay

The feasibility of the assay was determined using known qPCR-positive clinical samples, isolates, or gBlocks. For obtaining the analytical sensitivity of the primer pools, 10-fold serial dilutions of the positive clinical samples based on the cycle threshold (Ct) value or for the gBlocks, 100,000–100 copies of the gBlock segment, to find the ability of the assay to detect genomic sequence in dilutions. The primers were also validated for their specificity towards the pathogens under study by *in silico* analysis during the primer design process and by evaluating the sequences produced for each primer set using BLAST analysis with the Core nucleotide database and megablast (https://blast.ncbi.nlm.nih.gov/Blast.cgi). Sequences were considered accurate if ≥ 99% sequence length and identity matched the appropriate target.

### 2.4 Diagnostic sensitivity, specificity, and accuracy of the assay

The diagnostic sensitivity and specificity of the assay were tested using clinical samples submitted to the ADDL from different neurologic and reproductive cases of canines and from The Real-time PCR Research and Diagnostics Core Facility, the University of California, Davis (UC Davis). The samples from UC Davis were also used for blinded assay validation, which is described in the following section. In total, 76 samples were used for determining the diagnostic sensitivity, specificity, and accuracy of the developed test. The values were statistically calculated using the Clopper-Pearson method.

### 2.5 Reproducibility and blinded assay validation

Extracted nucleic acids from dogs previously tested by qPCR were purchased from The Real-time PCR Research and Diagnostics Core Facility, University of California, Davis, to validate the developed assay. These samples included extracts from whole blood, brain, CSF, nasal/conjunctival swabs, and lung (**Supplementary Table 2**). The testing was performed blinded by the ADDL, with results not provided from the laboratory at UC Davis until the testing was completed. For reproducibility testing, the tNGS assay and associated protocol were evaluated at an independent animal disease diagnostic laboratory (Pennsylvania animal Diagnostic Laboratory System- New Bolton Center (PADLS-NBC), University of Pennsylvania) with the same blinded sample set. Cohen’s kappa was calculated to determine the agreement between the results obtained with the tNGS assay from the two different laboratories.

## 3 Results

### 3.1 Feasibility and analytical specificity of tNGS assay

The synthesized panel of primers was validated for 34 different pathogens for their ability to amplify genomic segments of respective pathogens using Ion torrent sequencing technology. All pathogens included in the assay, except *Campylobacter jejuni* were sequenced in their respective clinical sample, bacterial isolate, or gBlock. Sequencing reads less than 100bp were ignored (considered false-priming). Based on the primer design, the size of the products is expected to be approximately 200 bp. The BLAST analysis of trimmed and aligned sequence reads obtained from TSS for individual samples confirmed the primer specificity towards the pathogen of interest. Although the sequencing of *Campylobacter jejuni-*positive clinical samples produced sequence reads that aligned with the pathogen, the BLAST analysis confirmed that the primers were amplifying nonspecific genomic fragments. The *Aspergillus* and *Actinomyces* primers also amplify additional organisms, but BLAST analysis will distinguish this. Additionally, one of the primer sets for *Streptococcus dysgalactiae subsp equisimilis* was not specific for this pathogen. However, a second primer set included in the assay was found to be specific for *S. dysgalactiae equisimilis*.

Furthermore, based on the BLAST analysis of the genomic sequences, we could characterize the Canine distemper virus and Canine parvovirus-2 clinical samples as America-3 genotype and genotype 2a, respectively. We could also identify the virulence genes such as cytotoxic necrotizing factors (cnf)1 and cnf 2 within the ExPEC. The details of the samples and their respective sources are described in **Table 1**.

### 3.2 Analytical sensitivity

The assay’s Limit of Detection (LOD) was calculated using serial dilutions of the pathogen-positive samples or gBlocks. The relative detection of the qPCR positive samples showed that the assay detected most of the pathogens at a limit comparable to Ct value of mid-30s (**Table 2**). However, certain discrepancies were observed in the limit of detection, especially for clinical samples of virus pathogens like Bluetongue virus (BTV), where multiple serotypes are present. A similar result was seen in the determination of the limit of detection where the primers for *Blastomyces dermatididis* could only detect the pathogen when at least 10,000 copies or more of the genomic fragments complementary to the primer included were present. For the other pathogens where gBlocks were used to calculate the LOD, the assay could identify the pathogen nucleic acid if 100–1000 copies of the same were present in the spiked samples (**Table 3**). Likewise, serotype variant gBlocks of BTV could be identified if 100–1000 copies were present.

**Table 2:** Relative limit of detection based on serial dilution of nucleic acids from qPCR-positive clinical samples.

Pathogen (qPCR target)	Relative Threshold Cycle (Ct), based on detection of gene target reads by tNGS in the sample at the detected Ct value
Canine distemper Virus (N gene)	37.99 (N gene and H gene reads detected)
Canine adenovirus-1 (E3 gene)	36.21 (E3 gene and ORF4 reads detected)
Eastern Equine Encephalitis Virus (NSP1 gene)	34.17 (RNA polymerase and E2 gene reads detected)
West Nile Virus (3’ noncoding region and E gene)	38.2 (M and E gene reads detected)
Bluetongue Virus serotype 10 (NS3 gene)	25 (NS1 and VP6 gene reads detected)
Rabies virus (N gene)	34.95 (N gene reads detected)
Canine Herpesvirus-1 (gB gene)	38 (gD and gB gene reads detected)
Canine Parvovirus-2 (VP2 gene)	37 (NS1 and VP2 gene reads detected)
*Toxoplasma gondii* (repetitive DNA sequence)	34.6 (repetitive DNA sequence reads detected)
*Leptospira* spp. (lipL32 gene)	34.9 (lipL32 gene reads detected)
*Mycoplasma* spp. (16S rRNA gene)	36.85 (16S-23S ribosomal RNA intergenic spacer region reads detected)
*Neospora caninum* (NC5 repeat element)	33.18 (NC5 repeat element reads detected)
*Pasteurella multocida* (KMT1 gene)	33.18 (Pm0762 and Pm1231 gene reads detected)

**Table 3:** Copy number-based detection limit of pathogens spiked with gBlocks representing targets within the pathogen genome.

Pathogen gBlock	Detection limit
Canine bocaparvovirus (Canine parvovirus-1)	1000
BTV- serotype 2	100
BTV serotype 11	1000
BTV serotype 17	100
*Brucella canis*	100
*Streptococcus dygalactiae subsp equisimilis*	100
*Borrelia burgdorferi*	100
*Blastomyces dermatitidis*	10,000
*Histoplasma capsulatum*	100
*Coccidioides immitis*	1000
*Aspergillus* spp.	1000
*Actinomyces* spp.	1000

### 3.3 Diagnostic sensitivity, specificity, and reproducibility of the assay

The diagnostic potential of the assay was measured based on the test’s ability to measure the results on clinical samples accurately (**Table 4**). Out of the 76 clinical samples tested during the study period, the assay identified 32/36 positive samples when compared to the gold standard qPCR results. Hence, a sensitivity of 88.89% at a 95% confidence interval (73.94%-96.89%) was calculated. Furthermore, among the 40 negative samples tested, the tNGS assay identified only one sample as a false positive, making the specificity 97.50% at a confidence interval of 95% (86.84- 99.94%) (**Table 4**; **Supplementary Table 1**). When comparing the accuracy of the developed tNGS with the standard qPCR assay, the former had an accuracy of 93.42%. The reproducibility testing performed at PADLS-NBC, University of Pennsylvania, using the primer pool provided by our laboratory, showed agreement in the results except for two samples. (**Supplementary Table 2**). Cohen’s Kappa was 0.77- this is considered a strong agreement; values greater than 0.75 or so may be taken to represent excellent agreement beyond chance.

**Table 4:** Comparison of positive and negative samples identified in tNGS relative to the gold standard test (qPCR).

		qPCR	
		Positive	Negative
tNGS	Positive	32	1
	Negative	4	39

## 4 Discussion

In canines, a variety of pathogens can contribute to neurological and reproductive diseases. Overlapping clinical signs, the potential for coinfection, and nonspecific symptoms make diagnosing neurological diseases particularly challenging. Currently, multiple diagnostic tests must be performed to reach a conclusion, yet in many cases, a definitive diagnosis remains elusive (Radaelli and Platt, 2002). Researchers have attempted to detect nucleic acids in cerebrospinal fluid (CSF) using broadly reactive PCR; however, viral nucleic acids were identified in only a few samples (Barber et al., 2022) or remained undetected (Jäderlund et al., 2009). Routine diagnostics often involve individual PCR tests, with or without sequencing, or multiplex PCRs to identify active infections. In case of reproductive pathogens, although most pathogens are endogenous in nature, pathogens previously not reported as reproductive pathogens are also considered as the cause of abortions (Akita et al., 1994; Wilbur et al., 1994). Additionally, only a few veterinary diagnostic laboratories offer canine neurological or reproductive panels for broad screening of different pathogens in cases of meningoencephalitis of unknown origin or abortion. This study aimed to develop a comprehensive assay capable of detecting neurological and reproductive disease agents in canines through a single test.

The advantage of using tNGS in clinical applications is attributed to the amount of data that it can generate in a single assay using a panel of primers targeting multiple pathogens, which is theoretically limitless. Also, it eliminates the difficulty of selecting the appropriate diagnostic test to use for a pathogen identification in case of diseases like neurological diseases, where the symptoms are nonspecific to a particular pathogen (Gunn-Moore, 2005), or in reproductive diseases, where most of the causative agents are endogenous (Watts et al., 1996). We successfully detected 33 pathogens that are clinically relevant to canine neurological and reproductive diseases.

The specificity problem encountered with *Aspergillus* in the assay was due to trying to target ITS regions of this organism. Some portions of the small and large ribosomal subunit RNAs were also accidentally targeted as part of the design. Removal of these regions would improve the specificity for this organism. A similar situation occurred for *Actinomyces*. We intended to target an *Actinomyces*-specific region of the 16S rRNA gene, but the final design had some primers that also amplified outside of this region. Using primers for genes specific for these organisms is better than using genes with universally conserved areas, like rRNA genes, for a tNGS assay. The target for *Campylobacter jejuni* was the hipO gene, which is specific for this organism, but unfortunately there were still off-target effects with the primers. There are some genetic changes in this gene between isolates, so that could have also affected assay sensitivity. Adding primers to account for differences in sequences and adding primers for an additional target would be ways to improve the assay for *C. jejuni* detection.

The sensitivity of the assay for most of the pathogens in this study was comparable to the sensitivity of qPCR for the same pathogens within the clinical range of Ct values 33–38 or approximately 100–1000 organisms per reaction, except for *Blastomyces dermatitidis*, which had a detection limit of approximately 10,000 copies. The target used for Blastomyces dermatitidis was the BAD-1 gene, which encodes an important conserved adhesion-promoting protein and is a virulence factor of *B. dermatitidis*. qPCR assays have been developed using this gene target, confirming its usefulness for molecular detection. However, this was the only target we included for *Blastomyces*, and as mentioned previously, adding another gene target would likely increase the sensitivity for this organism as well.

Certain pathogens like BTV serotype 10 were not within the expected range of 30s. We consider this either due to primer mismatch owing to the high variability and presence of several serotypes (Maclachlan, 2011) or due to the very low concentration of the fragmented virus genome in the sample. We designed gBlock for multiple BTV variants, and all those variants were detected in our assay in the range of 100–1000 copies of the segments we spiked, stating that the assay could identify the pathogen at low concentrations.

As shown in our earlier studies (Kattoor et al., 2022, 2024), the sensitivity is lower for tNGS assays compared to qPCR, but the number of samples used for both sensitivity and specificity were low and inclusion of a greater number of samples would provide a more accurate diagnostic sensitivity and specificity. Targeted NGS is more sensitive, faster, and cheaper in comparison to metagenomic NGS approaches, making it a better alternative when screening samples for known pathogens. The tNGS assay for syndromic testing can be completed from the clinical sample to the final data analysis in two days. Moreover, unlike the qPCR, tNGS could identify specific strains of certain viruses and toxin genes/virulence factors for most common reproductive pathogens such as genotyping for CDV and CPV-2 and virulence gene typing for *E.coli*.

We also tested our assay for reproducibility at PADLS-NBC, University of Pennsylvania, using the same protocol we developed. Out of the 18 samples, which included combinations of 8 different infectious agents, the labs disagreed in only 2, both of which involved detection of CDV. Although we could not conclusively determine the cause of the discrepancies, these were potentially due to repeated freeze-thaws of these samples (to explain negative CDV at PADLS-NBC but positive for CDV at the other two labs) or low level of organism RNA in the sample at the limit of detection and expected differences in replicates (for a sample that tested positive for CDV only at the ADDL). The latter sample was potentially a false-positive result at the ADDL, but the result was reproducible with repeat testing with the tNGS assay.

In conclusion, we developed a comprehensive tNGS panel for identifying pathogenic agents responsible for neurological and reproductive diseases in canines. Clinicians often face challenges due to the wide range of potential causative agents and the nonspecific nature of symptoms, necessitating multiple tests for accurate diagnosis. However, few veterinary laboratories offer neurological or abortion panels for these pathogens. In this study, we attempted to overcome this hurdle by developing a syndromic testing tNGS assay that targets 33 pathogens implicated in canine neurological and/or reproductive disease. The tNGS demonstrated a diagnostic sensitivity of 89% and specificity 98%, along with its ability to genotype and identify virulence factors for certain pathogens like CDV, CPV-2, and *E. coli*, increasing its use as an efficient diagnostic tool.

## Data Availability

The original contributions presented in the study are included in the article/**Supplementary Material**. Further inquiries can be directed to the corresponding author.

## Supplementary material

**Supplementary Tables 1** and **2** for the article are available as a separate document. FASTA and BED files are available as **Supplementary Material**, as separate documents.
