# Supplementary material for: A multiplex targeted NGS panel for identifying pathogens in canine neurological and reproductive diseases
Source: Front Cell Infect Microbiol. 2025 Aug 1;15:1591145. doi: 10.3389/fcimb.2025.1591145 (PMC12355606; doi:10.3389/fcimb.2025.1591145)
Supplement: Supplementary file 1 [file Table1.docx]

Supplementary Material

# Supplementary Tables

**Supplementary Table 1:** Details of samples tested with developed targeted next-generation sequencing (tNGS) assay along with the gold standard test available, qPCR or aerobic culturing.

| **Sample ID** | **Sample Type** | **qPCR** | **Aerobic culture** | **tNGS assay** |
| --- | --- | --- | --- | --- |
| 1 | CSF | CDV Negative | Not performed | No pathogens detected |
| 2 | CSF | CDV Negative | Not performed | No pathogens detected |
| 3 | CSF | CDV Negative | Not performed | No pathogens detected |
| 4 | CSF | CDV Negative | Not performed | No pathogens detected |
| 5 | CSF | CDV Negative | Not performed | No pathogens detected |
| 6 | CSF | CDV Negative | Not performed | No pathogens detected |
| 7 | CSF | CDV Negative | Not performed | No pathogens detected |
| 8 | CSF | CDV Negative | Not performed | No pathogens detected |
| 9 | CSF | CDV Negative | Not performed | No pathogens detected |
| 10 | CSF | CDV Negative | Not performed | No pathogens detected |
| 11 | CSF | CDV Ct 34.15 | Not performed | CDV- Am-5 genotype detected |
| 12 | CSF | CDV Negative | Not performed | No pathogens detected |
| 13 | CSF | CDV Negative | Not performed | No pathogens detected |
| 14 | CSF | CDV Negative | Not performed | No pathogens detected |
| 15 | CSF | CDV Negative | Not performed | No pathogens detected |
| 16 | CSF | CDV Negative | Not performed | No pathogens detected |
| 17 | CSF | CDV Negative | Not performed | No pathogens detected |
| 18 | CSF | CDV Negative | Not performed | No pathogens detected |
| 19 | CSF | CDV Negative | Not performed | No pathogens detected |
| 20 | CSF | CDV Negative | Not performed | No pathogens detected |
| 21 | CSF | CDV Negative | Not performed | No pathogens detected |
| 22 | CSF | CDV Negative | Not performed | No pathogens detected |
| A24-21671* #5 | Nasal swab | CDV Ct 21.51 | Not performed | CDV Am-5, Pneumovirus, *Bordetella bronchiseptica* |
| A24-21671* #7 | Nasal swab | CDV Ct 24.83 | Not performed | CDV Am-5, Pneumovirus, *Bordetella bronchiseptica* |
| A24-21671* #9 | Nasal swab | CDV Ct 19.46 | Not performed | CDV Am-5, Pneumovirus, *Bordetella bronchiseptica* |
| A24-21671* #11 | Nasal swab | CDV Ct 30.43 | Not performed | CDV Am-5, *Bordetella bronchiseptica* |
| A24-21671* #12 | Nasal swab | CDV Ct 23.2 | Not performed | CDV Am-5, Pneumovirus, Canine parainfluenza |
| A24-15095 | Brain tissue | CDV Ct 21.75 | Not performed | CDV- could not type |
| A24-15095 | Brain tissue nucleic acids spiked with *Histoplasma capsulatum* gblock (approximately 10,000 copies) | CDV Ct 21.75 | Not performed | CDV,  *Histoplasma capsulatum* |
| A24-21925 | Brain tissue nucleic acids spiked with *Blastomyces dermatitidis* gblock (approximately 10,000 copies) | CDV, *Neospora caninum*, *Toxoplasma gondii* all negative | Not performed | *Blastomyces dermatitidis* |
| A25-12075 | Brain tissue nucleic acids spiked with *Coccidioides immitis* gblock (approximately 10,000 copies) | CDV negative | No growth | *Coccidioides immitis* |
| A24-15659 | Brain tissue nucleic acids spiked with *Histoplasma capsulatum* gblock (approximately 10,000 copies) | CDV negative | *E. coli* (considered contamination) | *Histoplasma capsulatum,*  *E. coli* eae |
| A25-5628 | Brain tissue nucleic acids spiked with *Blastomyces dermatitidis* gblock (approximately 10,000 copies) | Rabies PCR and DFAT negative | Not performed | *Blastomyces dermatitidis* |
| A21-10117 | Fetal tissue nucleic acids spiked with CPV type 1 (Carnivore bocaparvovirus 1) gblocks (approximately 10,000 copies) | *Brucella sp.*, *Toxoplasma gondii*, Canine herpesvirus, CDV, and Adenovirus type1 all negative | Negative | CPV type 1 |
| A21-11228 | Fetal tissues spiked with *Streptococcus equi* zooepidemicus isolate | *Brucella sp.*, *Toxoplasma gondii*, Canine herpesvirus, CDV, and Adenovirus type1 all negative | Negative | *Streptococcus equi zooepidemicus* |
| A21-13510 | Fetal tissue nucleic acids spiked with CAV-1 gblocks (approximately 10,000 copies) | *Brucella sp.*, *Toxoplasma gondii*, Canine herpesvirus, CDV, and Adenovirus type1 all negative (prior to spike) | Negative | Canine Adenovirus 1 |
| A20-11686 | Fetal tissues spiked with *E. coli* ExPEC isolate (confirmed by WGS) | *Brucella sp.*, *Toxoplasma gondii*, Canine herpesvirus, CDV, and Adenovirus type1 all negative | Negative (prior to spike) | *E. coli* cnf 1 |
| A23-3217 | Fetal tissues spiked with *E. coli* ExPEC isolate (confirmed by WGS) | *Brucella sp.*, *Toxoplasma gondii*, Canine herpesvirus, CDV, and Adenovirus type1 all negative | Negative (prior to spike) | *E. coli* cnf 1 |
| A19-9983 | Fetal tissue pool | Canine herpesvirus positive  *Brucella* sp., *Toxoplasma gondii*, CDV, and Adenovirus type1 all negative | Negative | Canine herpesvirus detected |
| A22-4651 | Fetal tissue pool | *Brucella* sp., *Toxoplasma gondii*, Canine herpesvirus, CDV, and Adenovirus type1 all negative | Negative | No pathogens detected |
| A22-6938 | Fetal tissue pool | *Brucella* sp., *Toxoplasma gondii*, Canine herpesvirus, CDV, and Adenovirus type1 all negative | Negative | No pathogens detected |
| A23-488 | Fetal tissue pool | *Brucella* sp., *Toxoplasma gondii*, Canine herpesvirus, CDV, and Adenovirus type1 all negative | Negative | No pathogens detected |
| A23-1399 | Fetal tissue pool | *Brucella* sp., *Toxoplasma gondii*, Canine herpesvirus, CDV, and Adenovirus type1 all negative | Negative | No pathogens detected |
| A22-14246 | Fetal tissue pool | *Brucella* sp., *Toxoplasma gondii*, Canine herpesvirus, CDV, and Adenovirus type1 all negative | Negative | No pathogens detected |
| A22-17611 | Fetal tissue pool | *Brucella* sp., *Toxoplasma gondii*, Canine herpesvirus, CDV, and Adenovirus type1 all negative | Negative | No pathogens detected |
| A23-8295 | Fetal tissue pool | *Brucella* sp., *Toxoplasma gondii*, Canine herpesvirus, CDV, and Adenovirus type1 all negative | Negative | No pathogens detected |
| A23-9597 | Fetal tissue pool | *Brucella* sp., *Toxoplasma gondii*, Canine herpesvirus, CDV, and Adenovirus type1 all negative | Negative | No pathogens detected |
| S22-1245 | Neonatal tissue pool | *Brucella* sp., *Toxoplasma gondii*, Canine herpesvirus, CDV, and Adenovirus type1 all negative | *Staphylococcus pseudintermedius* from liver and spleen (neonatal septicemia diagnosed) | *Staphylococcus pseudintermedius* detected |
| A22-10404 | Fetal tissue pool | *Brucella* sp., *Toxoplasma gondii*, Canine herpesvirus, CDV, and Adenovirus type1 all negative | Negative | No pathogens detected |
| A22-2784 | Fetal tissue pool | *Brucella* sp., *Toxoplasma gondii*, Canine herpesvirus, CDV, and Adenovirus type1 all negative | Negative | No pathogens detected |
| A21-14810 | Neonatal tissue pool | *Brucella* sp., *Toxoplasma gondii*, Canine herpesvirus, CDV, and Adenovirus type1 all negative | *Streptococcus canis* and *E coli* cultured (Hemorrhagic bronchopneumonia and necrotizing fasciitis diagnosed) | *Streptococcus canis* and *E coli* FocG gene detected- associated with adhesion to epithelial and endothelial cells |
| A21-17489 | Fetal tissue pool | *Brucella* sp., *Toxoplasma gondii*, Canine herpesvirus, CDV, and Adenovirus type1 all negative | *E coli* from fetal spleen and liver | *E coli* cnf 1 and hlyD (both associated with ExPEC) |
| A20-5218 | Fetal tissue pool | *Brucella* sp., *Toxoplasma gondii*, Canine herpesvirus, CDV, and Adenovirus type1 all negative | *Staphylococcus pseudintermedius* (placentitis and sepsis) | *Staphylococcus pseudintermedius* detected |
| A20-2293 | Fetal tissue pool | *Brucella* sp., *Toxoplasma gondii*, Canine herpesvirus, CDV, and Adenovirus type1 all negative | Negative | No pathogens detected |
| A24-7349 | Neonatal tissue pool | *Brucella* sp., *Toxoplasma gondii*, Canine herpesvirus, CDV, and Adenovirus type1 all negative | Negative | No pathogens detected |
| A24-15068 | Fetal tissue pool | *Brucella* sp., *Toxoplasma gondii*, Canine herpesvirus, CDV, and Adenovirus type1 all negative | Negative | No pathogens detected |
| A20-8076 | Fetal tissue pool | *Brucella* sp., *Toxoplasma gondii*, Canine herpesvirus, CDV, and Adenovirus type1 all negative | *Listeria monocytogenes* | *Listeria monocytogenes* detected |
| A20-6946 | Uterus | *Brucella* spp. PCR negative | Not tested | No pathogens detected |

CDV- Canine distemper virus

* Samples were from an outbreak in an animal shelter; the animals had a mix of respiratory and neurological signs. Nasal swabs were collected from live animals for testing.

**Supplementary Table 2:** Comparison of results between in-house and independent laboratory tNGS results. A blinded sample pool of 18 samples, including a combination of 8 different pathogens received from UC Davis, were verified using a standardized qPCR panel for their respective pathogens.

| **Sample ID (samples were nucleic acids previously extracted at UC Davis)** | **UC Davis qPCR** | **tNGS (in-house)** | **tNGS (Independent Laboratory)** |
| --- | --- | --- | --- |
| Neuro 1 whole blood | CDV Ct 20.8; *Toxoplasma* Ct 39- negative by our assay | CDV America 3 genotype; no *Toxoplasma* reads | CDV, no *Toxoplasma* detected |
| Neuro 2 brain tissue | CDV Ct 29.27; *Toxoplasma* Ct 39.85- negative by our assay | CDV America 3 genotype; CPV 2c; Alphacoronavirus-1 (likely breakdown of BBB) | CPV 2, Alphacoronavirus-1, **NO CDV detected** |
| Neuro 3 CSF | *Neospora* 31.34; *Toxoplasma* 39.9- negative by our assay | *Neospora caninum*; no *Toxoplasma* detected | *Neospora caninum*, no Toxoplasma detected |
| Neuro 4 CSF | *Neospora* Ct 36.86- Ct 35.3 by our assay | No specific pathogens detected | No specific pathogens detected |
| Neuro 5 CSF | CDV Ct 27.45 | CDV America 3 | CDV |
| Neuro 6 brain tissue | CDV Ct 29.24 | CDV America 3 | CDV |
| Neuro 7 pool CSF and whole blood | *Neospora* 28.23; *Toxoplasma* 34.45- Ct 34.87 by our assay | *Neospora caninum;* No *Toxoplasma gondii* reads detected | *Neospora caninum*, no *Toxoplasma* detected |
| Respiratory 1 nasal swab | CHV Ct 19.48; CDV Ct 22.91 | CHV and CDV- America 3 genotype | CHV and CDV |
| Respiratory 2 nasal swab | No respiratory pathogens detected | CDV- America 3 | **No pathogens detected** |
| Respiratory 3 nasal swab | No respiratory pathogens detected | No specific pathogens- normal flora | No pathogens detected |
| Respiratory 4 conjunctival swab | CHV Ct 26.96 | CHV | CHV |
| Respiratory 5 nasal swab | CHV Ct 31.52; *Streptococcus equi zooepidemicus* Ct 35.98 | CHV; *Mycoplasma cynos*; No *Streptococcus equi zooepidemicus* detected | CHV, *Mycoplasma cynos*,, No *Streptococcus equi zooepidemicus* detected |
| Respiratory 6 nasal swab | No respiratory pathogens detected | No significant pathogens detected- normal flora | No significant pathogens |
| Respiratory 7 lung tissue | CHV Ct 15.98 | CHV | CHV |
| Respiratory 8 nasal swab | No respiratory pathogens detected | No specific pathogens detected- normal flora | No specific pathogens |
| Respiratory 9 lung tissue | *Streptococcus equi zooepidemicus* Ct 21.21 | *Streptococcus equi zooepideimicus* | *Streptococcus* sp. |
| Respiratory 10 nasal swab | *Streptococcus zooepidemicus* Ct 28.8 | No specific pathogens  no *S. zooepidemicus* detected | No specific pathogens detected- no *S. zooepidemicus* detected |
| Respiratory 11 nasal swab | No respiratory pathogens detected | *Mycoplasma canis* detected as well as other normal flora | No specific pathogens |

CDV- Canine distemper virus; CPV- Canine parvovirus; CHV- Canine herpesvirus
